# Supplementary material for: Chemically defined and xeno-free culture condition for human extended pluripotent stem cells
Source: Nat Commun. 2021 May 21;12:3017. doi: 10.1038/s41467-021-23320-8 (PMC8139978; doi:10.1038/s41467-021-23320-8)
Supplement: Supplementary file 1 — Supplementary Information [file 41467_2021_23320_MOESM1_ESM.pdf]

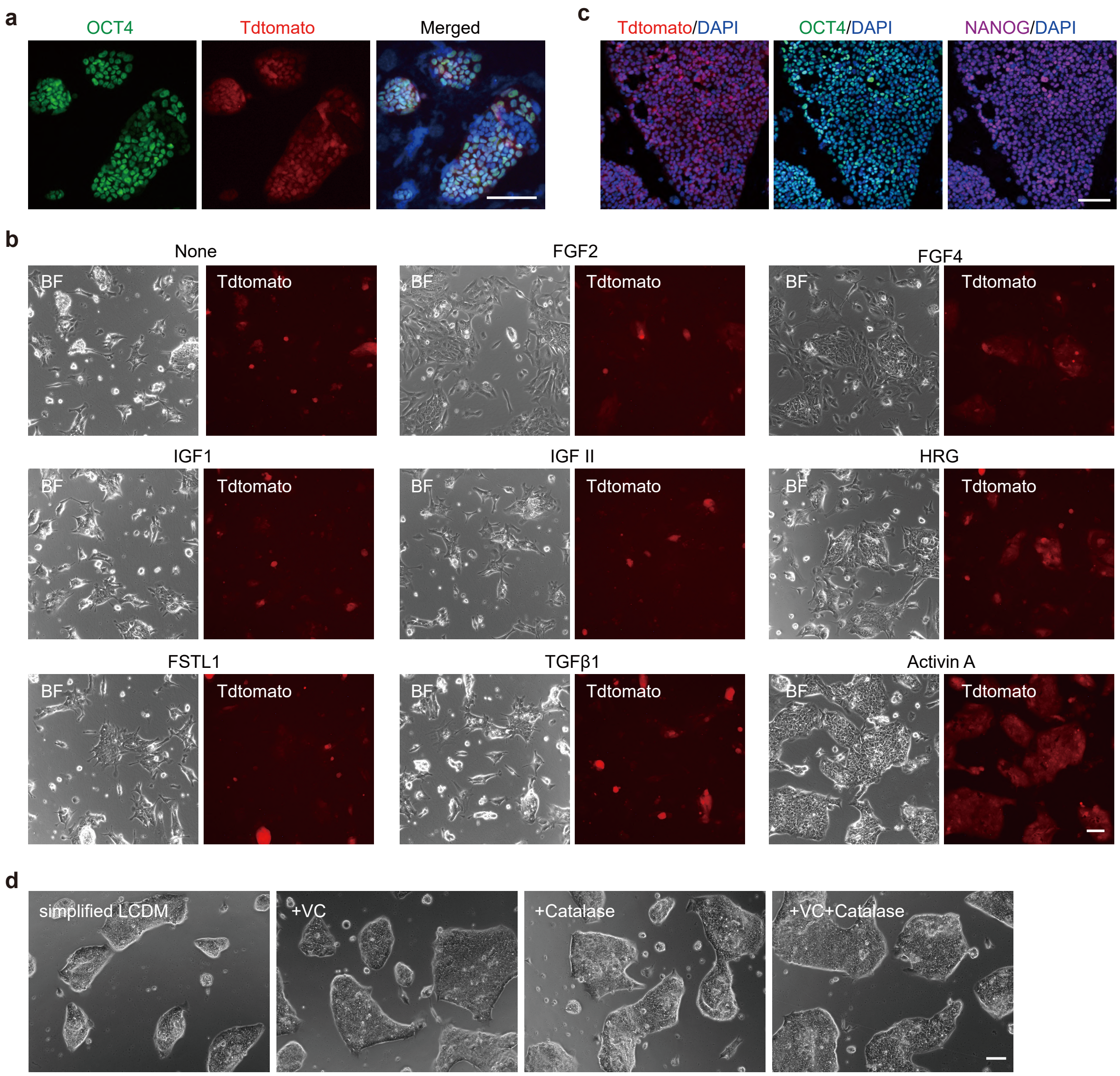

Supplementary Fig. 1. Identification of factors that are important for culturing human EPS cells under the feeder-free condition.

a OCT4 and Tdtomato expression in H1-EPS cells with the knocked-in OCT4-Tdtomato reporter (OT H1-EPS). **b** Representative images showing morphologies and OCT4-Tdtomato expression in OCT4-Tdtomato H1-EPS cells under different factor treatments. Cells were cultured without feeders for 2 passages. BF, bright field. None, LCDM medium. **c** Representative images showing immuno-staining of OCT4 and NANOG expression in OT H1-EPS cells with Activin A treatment under the feeder-free condition. **d** Representative images showing the morphologies of feeder-free H1-EPS cells cultured with vitamin C, catalase or their combination. VC, vitamin C. Simplified LCDM, N2 and B27 are replaced by ITSX, and Activin A is also added. Scale bars, 100 μm. For representative images shown in (a-d), similar images were obtained in at least 3 independent experiments. Experiments in (a-d) were all independently repeated at least three times with similar results.

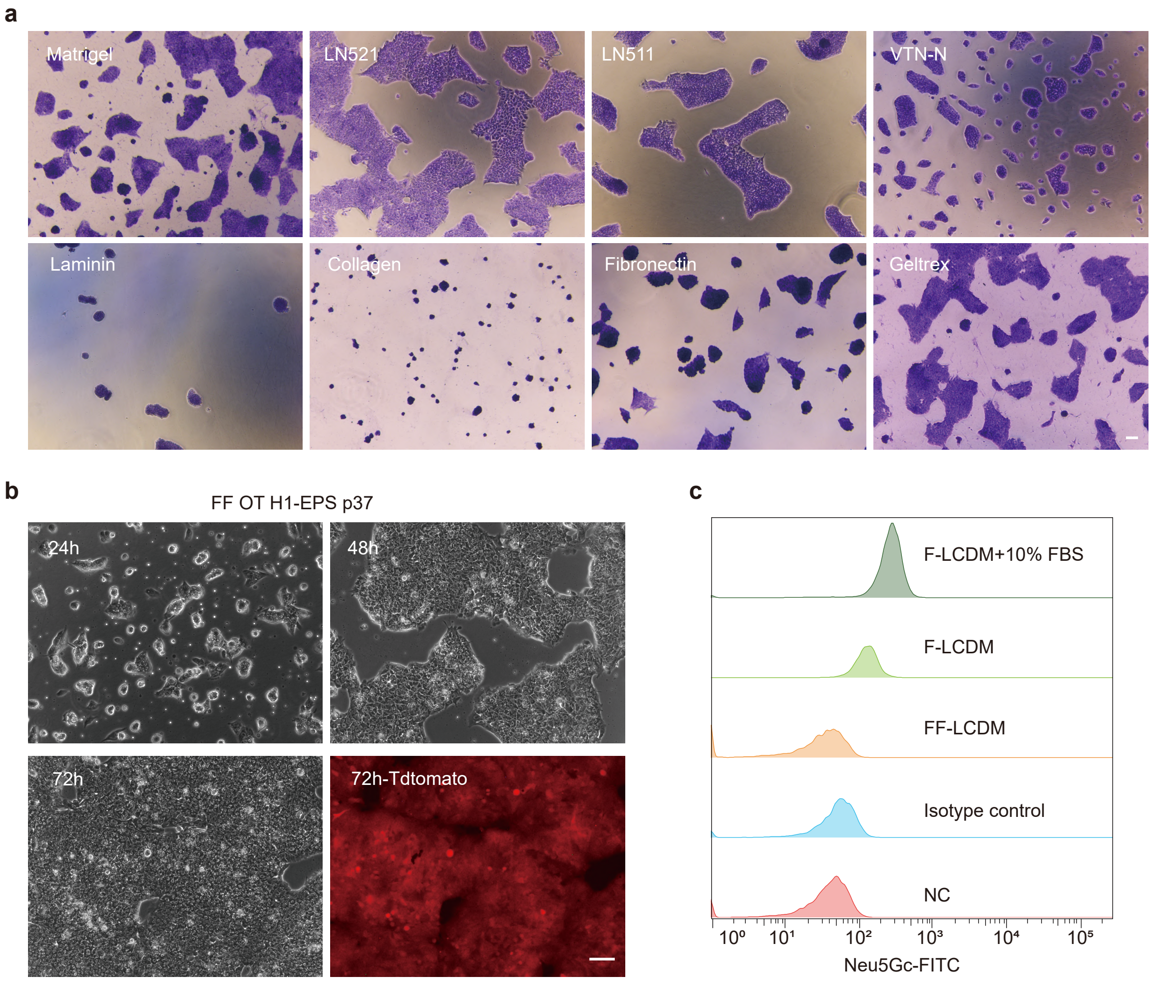

Supplementary Fig. 2. Laminin 521 supports xeno-free culturing of human EPS cells.
a Representative Violet staining showing the survival of H1-EPS cells cultured on different matrix proteins at 72 hours after seeding. **b** Representative images showing morphological dynamics of OT H1-EPS cells cultured on Laminin 521 at 24, 48 and 72 hours after seeding. OCT4-tdtomato expression was also shown at 72 hours after seeding. For representative images shown in (a-b), similar images were obtained in at least 3 independent experiments. **c** Representative FACS analysis of Neu5Gc expression in hEPS cell cultures under different conditions. ES1-EPS cells were used. F-LCDM, hEPS cells are cultured on feeders using the LCDM medium. F-LCDM+10% FBS, hEPS cells are cultured on feeders using the LCDM medium plus 10% FBS. FF-LCDM, hEPS cells are cultured without feeders using the xeno-free LCDM medium. Isotype control, isotype antibody is used to replace anti-Neu5GC antibody, and hEPS cells are cultured without feeders using the xeno-free LCDM medium. NC, no antibody is used, and hEPS cells are cultured without feeders using the xeno-free LCDM medium. Scale bars, 100 μm. Experiments in (a-c) were all independently repeated at least three times with similar results.

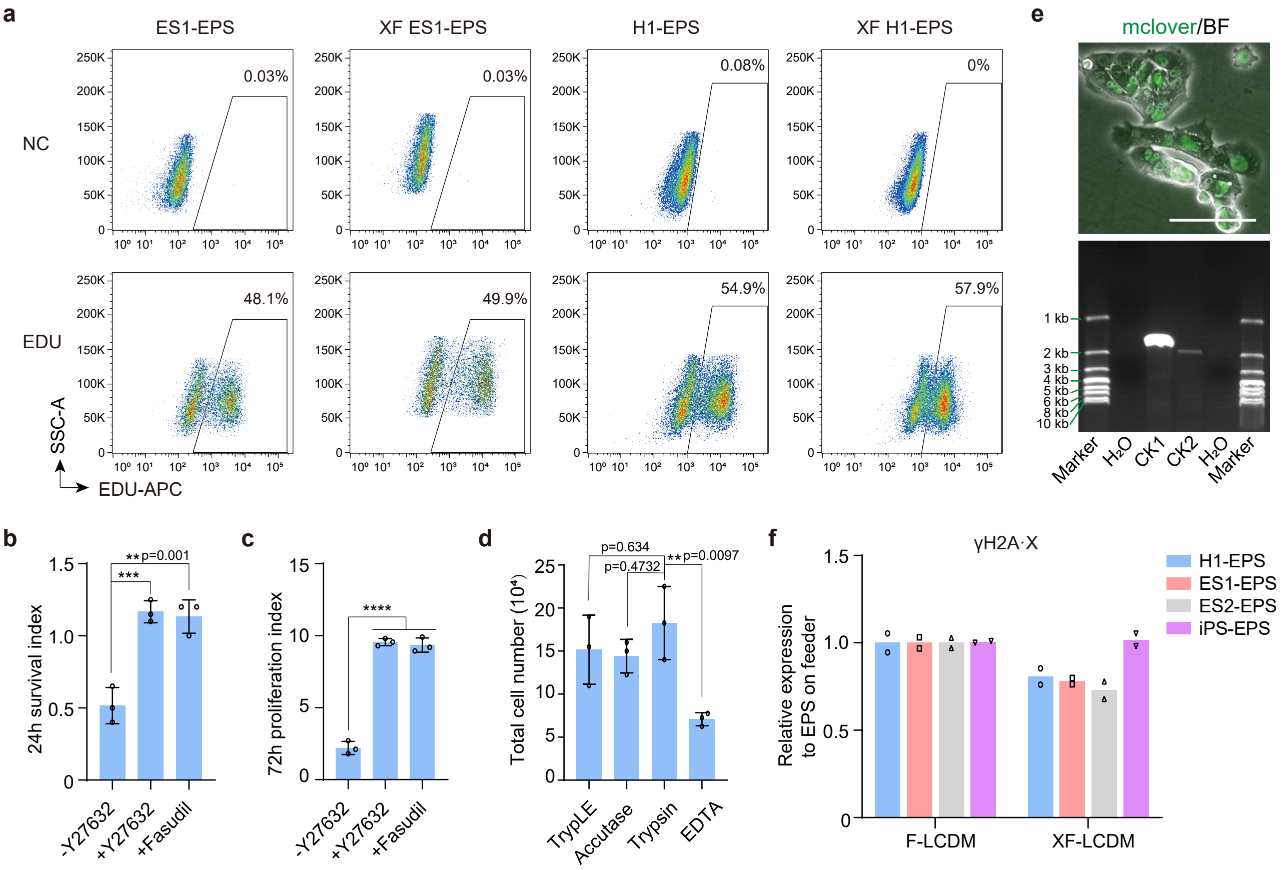

Supplementary Fig. 3.Further characterization of xeno-free human EPS cells.
a FACS analysis of the percentages of EDU+ cells in feeder-cultured and xeno-free hEPS cells. Representative images of FACS analysis are shown. **b-c** Survival and proliferation of xeno-free hEPS cells under Rock-inhibitor treatment. XF H1-EPS cells were used. The concentration of Y27632 and Fasudil was 5 μM. Index represents the cell number at a specific time point divided by the number of seeding cells. For b, c, n = 3 biologically independent samples. **d** Dissociation of xeno-free hEPS cells using different dissociation reagents. XF ES1-EPS cells were used. Cells were cultured in 24-well plates. Cell numbers are calculated after dissociation. n = 4 biologically independent samples. **e** Gene targeting of mclover reporter into the AAVS1 locus in xeno-free hEPS cells. XF ES1-EPS cells were used. Upper panel, representative image showing mclover expression in XF ES1-EPS cells. Similar images were obtained in at least 2 independent experiments. BF, bright field. Lower panel, genome PCR validates the insertion of mclover reporter into the AAVS1 locus in XF ES1-EPS cells. CK1 and CK2, primers that are specific to detect the homogenous recombination occurred at 3'arm and 5'arm of the targeting vector respectively. **f** FACS analysis of the presence of γ-H2AX signals in feeder-cultured and xeno-free hEPS cells. n = 2 biologically independent samples. F-LCDM, hEPS cells cultured on feeders; XF-LCDM, xeno-free hEPS cells. Error bars, mean ± SD. All differences between means with $P<0.01$ are indicated. **, $P < 0.01$; ***, $P<0.001$; ****, $P<0.0001$. Statistical significance was analyzed using one-way ANOVA with Tukey multiple comparison test. (b-d). Scale bars, 100 μm. Experiments in (a-f) were all independently repeated at least three times with similar results.

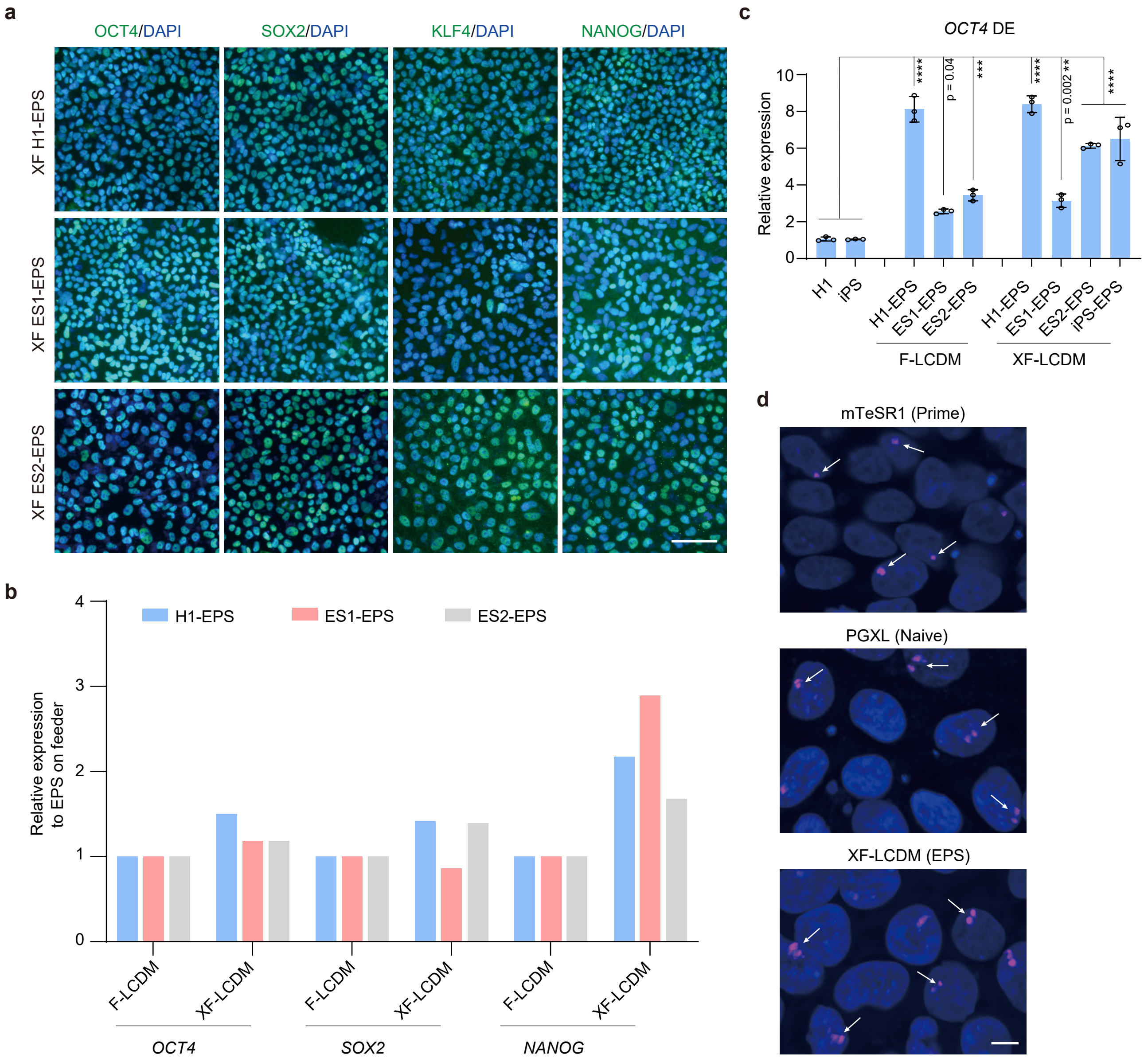

Supplementary Fig. 4. Further characterization of the molecular features of xeno-free human EPS cells.
a Representative immuno-staining images showing pluripotent marker gene expression in xeno-free hEPS cells. Scale bar, 100 μm. Similar images were obtained in at least 3 independent experiments. **b** Q-PCR analysis of pluripotent marker gene expression in xeno-free hEPS cells. n = 3 technically independent samples. **c** Predominant utilization of OCT4 distal enhancer element in feeder-cultured and xeno-free hEPS cells. Primed human PSCs (H1 and iPS) were used as controls. Human OCT4 transcriptional regulation is evaluated by the activity of distal enhancer reporter gene using the luciferase reporter assay in the indicated cell lines. Baseline activity was analyzed by transfection with an empty vector. n = 3 biologically independent samples. Error bars, mean ± SD. All differences between means with $P<0.01$ are indicated. **, $P < 0.01$; ***, $P<0.001$; ****, $P<0.0001$. Statistical significance was analyzed using one-way ANOVA with Tukey multiple comparison test. **d** Representative RNA-FISH images of different kind of pluripotent stem cells. Scale bar, 8 μm. F-LCDM, hEPS cells cultured on feeders; XF-LCDM, xeno-free hEPS cells. Similar images were obtained in at least 3 independent experiments. Experiments in (a-d) were all independently repeated at least three times with similar results.

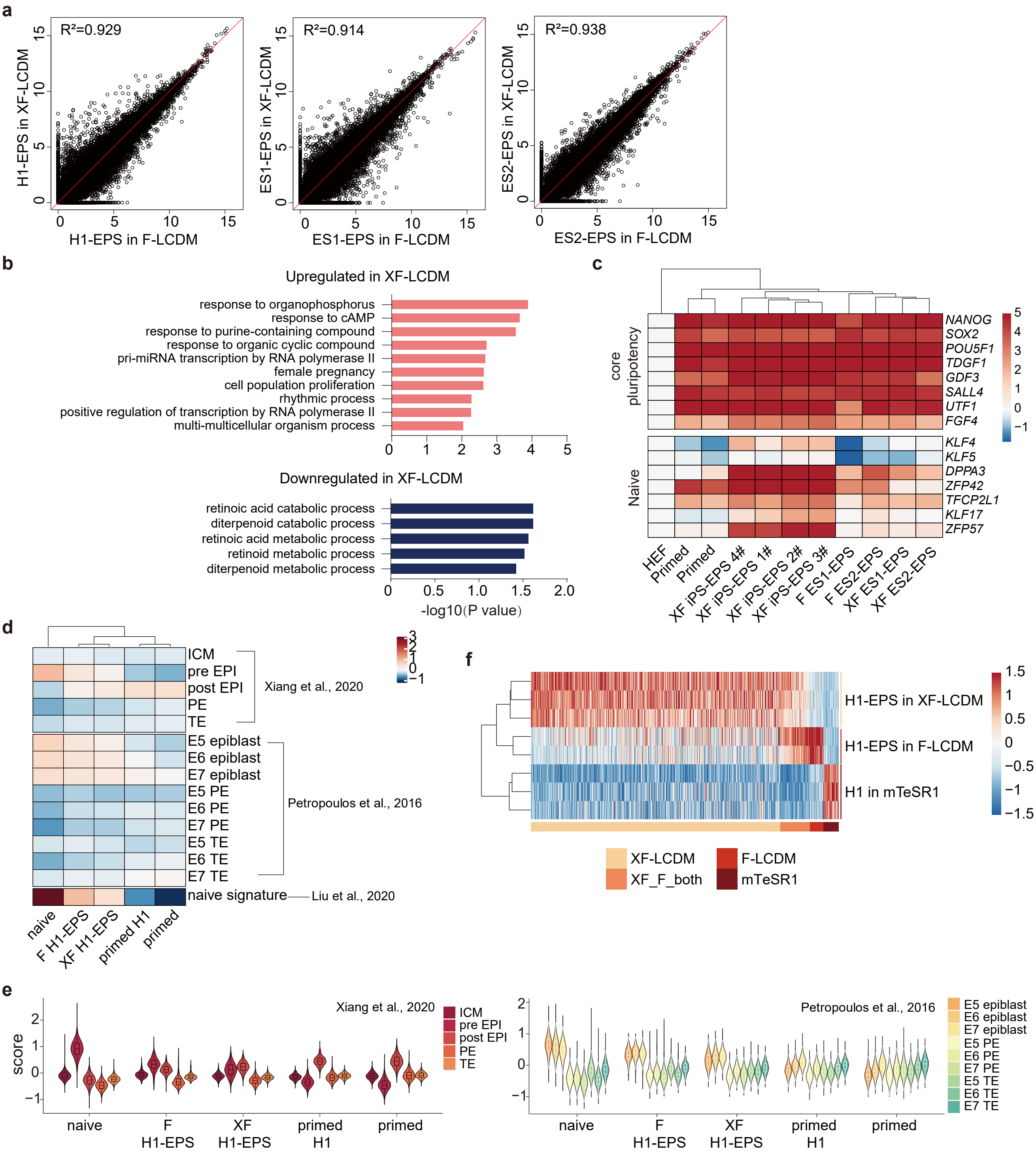

Supplementary Fig. 5. Transcriptomic analysis of xeno-free human EPS cells.

a Analysis of global gene expression correlation between feeder-cultured and xeno-free hEPS cells. **b** Enriched gene ontology (GO) terms for biological process in XF-LCDM differentially expressed genes. Red and blue bars indicate up- and down-regulated genes in XF-LCDM compared to F-LCDM, respectively. **c** Heatmaps of selected pluripotency related genes in human HEF, primed human PSCs (H1), feeder cultured EPS cells and xeno free EPS cells. **d** Heatmaps showing expression enrichment of gene signatures for different lineages from pre-implantation human embryos (E5/E6/E7) in xeno-free and feeder-cultured hEPS cells. Single cell RNA-seq data for xeno-free, feeder-cultured hEPS cells and primed H1 cells were used. As the control, single RNA seq data for naïve and primed hPSCs from Liu et al., 2020 were re-analyzed[1]. Gene signatures for TE/EPI/PE were defined in the study performed by Petropoulos et al., 2016[2]. Gene signatures for ICM, pre- and post- implantation EPI and post- implantation PE/TE were defined in the study performed by Xiang et al., 2020[3]. Naïve signatures were defined in the study performed by Liu et al., 2020[1]. **e** Violin plots showing the gene signature scores distribution of different cell types. Single cell RNA-seq data were used. **f** Heatmaps showing the specific ATAC-seq peaks of hEPS cells cultured in XF-LCDM and F-LCDM, and primed hPSCs cultured in mTeSR1. For boxplots, the center line corresponds to the median, and the lower and upper hinges correspond to the first and third quartiles (the 25th and 75th percentiles). The upper and lower whisker extends from the hinge to the largest and smallest value no further than 1.5 * IQR from the hinge, respectively. (IQR, inter-quartile range, the distance between the first and third quartiles).

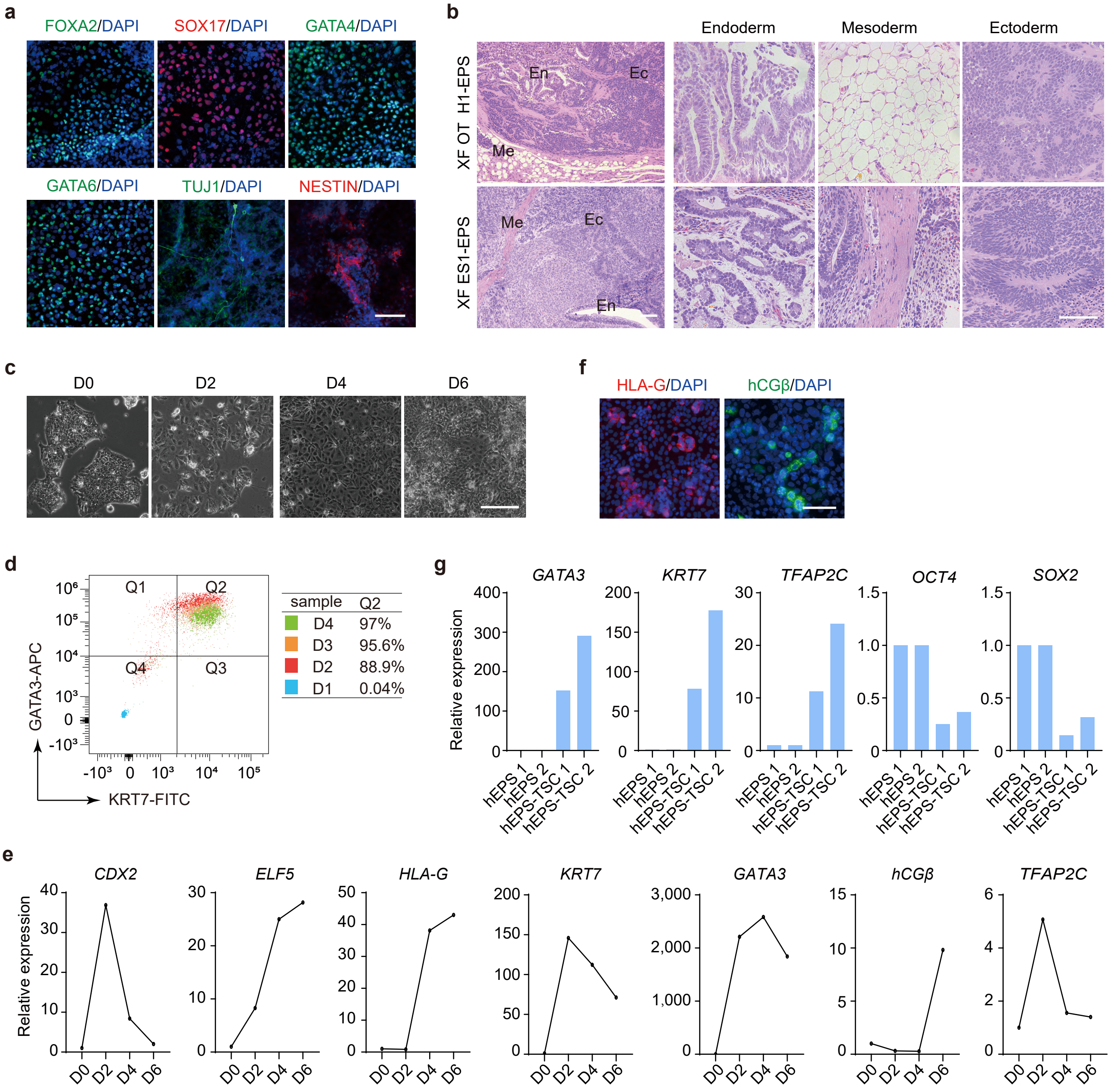

Supplementary Fig. 6. Further characterization of the in vitro differentiation potentials of xeno-free human EPS cells.

a Representative immune-staining images showing differentiated marker expression in xeno-free hEPS derivatives after EB differentiation. XF ES1-EPS cells were used. Similar images were obtained in at least 3 independent experiments. **b** Representative images showing histological analysis of teratomas generated from xeno-free hEPS cells. Similar images were obtained in at least 3 independent experiments. **c** Representative images showing morphological changes of XF-hEPS cells during their differentiation to trophoblast cells. XF H1-EPS cells were used. Similar images were obtained in at least 3 independent experiments. **d** Representative images showing FACS analysis of KRT7 and GATA3 expression in xeno-free hEPS derivatives during trophoblast differentiation. NC, without secondary fluorescent antibody. XF H1-EPS cells were used. **e** Q-PCR analysis of dynamics of trophoblast marker gene expression during trophoblast differentiation of xeno-free hEPS cells. XF H1-EPS cells were used. n = 2 technically independent samples. Gene expression at different time points is normalized to that at D0. **f** Representative immuno-staining images showing the expression of HLA-G and hCGβ in xeno-free hEPS derived trophoblast-like cells. XF H1-EPS cells were used. Similar images were obtained in at least 3 independent experiments. **g** Q-PCR analysis of trophoblast and pluripotent marker gene expression of xeno-free hEPS derived TSC like cells. n = 2 technically independent samples. Gene expression at different time points is normalized to XF-EPS cells. XF H1-EPS and XF ES1-EPS cells were used. Scale bars, 100 μm. Experiments in (a-g) were all independently repeated at least three times with similar results.

a

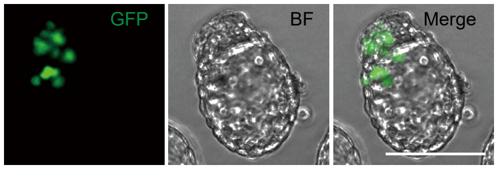

Cell lines	Recovered embryos	Contributed into both TE & ICM
XF ES1-EPS	42	26
XF ES2-EPS	24	10

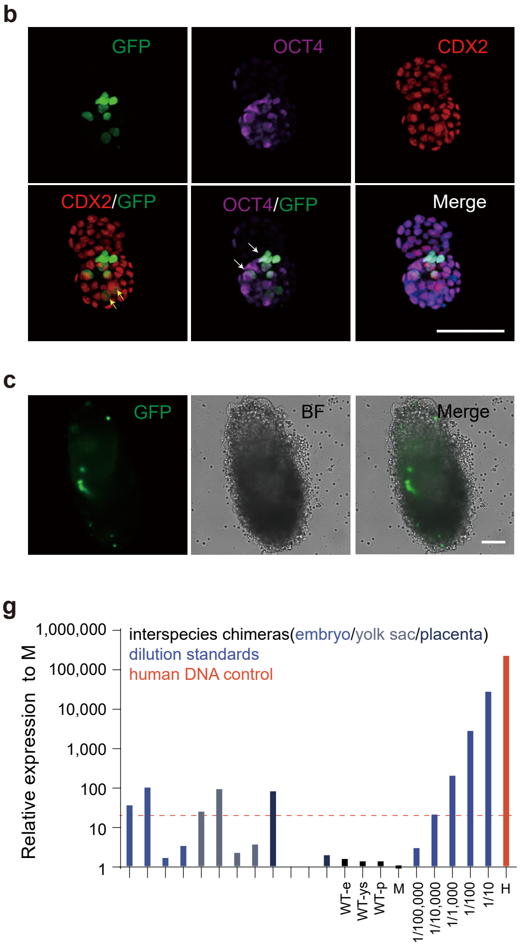

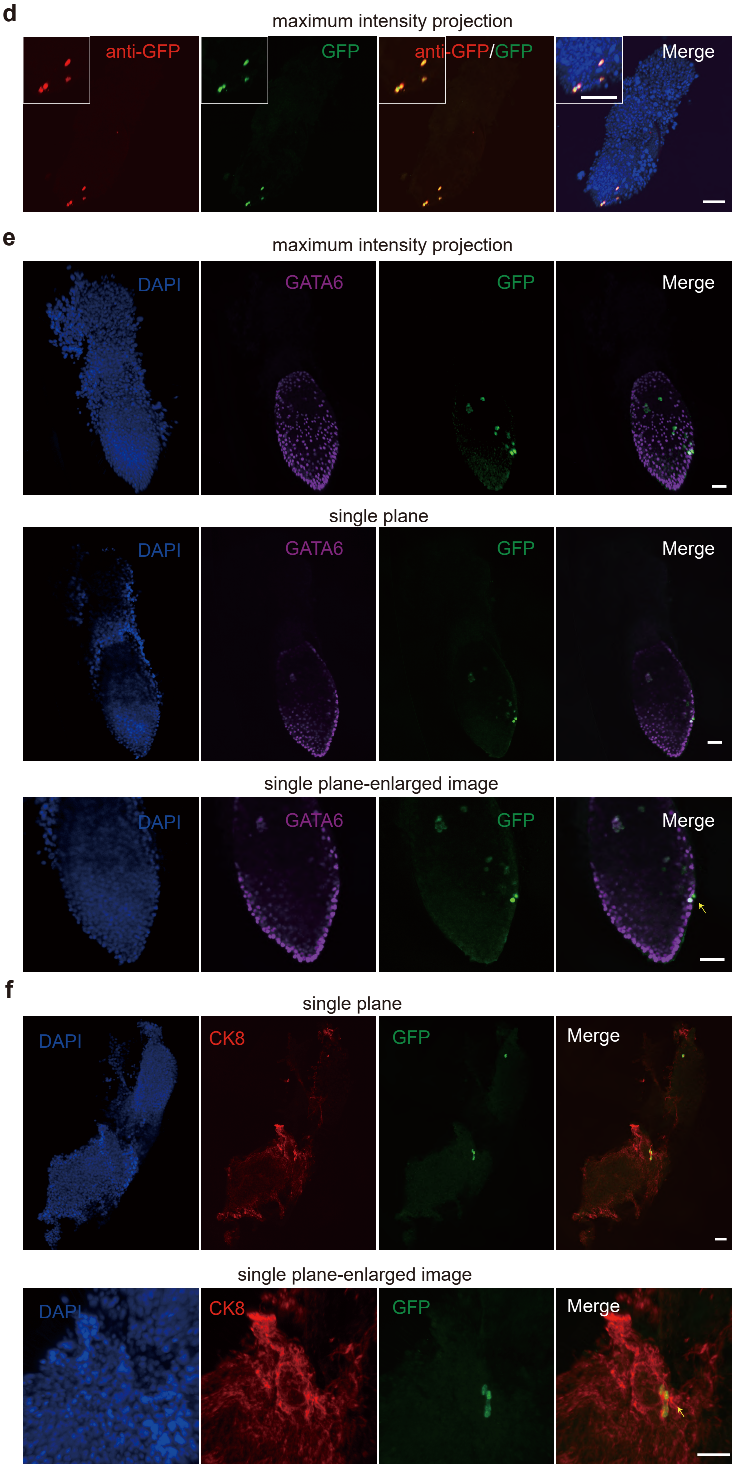

Supplementary Fig. 7. Characterization of the in vivo differentiation potentials of xeno-free human EPS cells.
a Chimerism of xeno-free hEPS cells in E4.5 mouse blastocysts. Representative images of chimerism were shown. A summary of the chimeric experiment was also shown. Scale bars, 100 μm. Similar images were obtained in at least 5 independent experiments. **b** Representative images of immunofluorescent staining showing xeno-free hEPS cells in E4.5 mouse blastocysts. Scale bars, 100 μm. Similar images were obtained in at least 5 independent experiments. **c** Representative images showing chimerism of xeno-free hEPS cells in E6.5 mouse conceptuses. mclover-XF ES1-EPS cells were used. Scale bar, 100 μm. Similar images were obtained in at least 5 independent experiments. **d** Immunofluorescent staining of E6.5 chimeric embryos showing the mclover-XF EPS cells (GFP) co-stained with anti-GFP antibody. Scale bar, 100 μm. Similar images were obtained in at least 3 independent experiments. **e-f** Immunofluorescent staining of E6.5 chimeric embryos showing lineage contributions of xeno-free hEPS derivatives to primitive endoderm derivatives or trophoblast lineages. mclover-XF ES1-EPS cells were used. Embryos were stained for GATA6 (primitive endoderm derivatives), CK8 (trophoblast compartment) and mclover (GFP). Scale bars, 50 μm. Similar images were obtained in at least 5 independent experiments. **g** Quantitative PCR analysis for human mitochondrial DNA indicated the presence of human cells in mouse embryos, yolk sacs and placentas at E10.5 following injection of hEPS cells in the 8-cell embryos. n = 2 technically independent samples. Experiments in (a-g) were all independently repeated at least three times with similar results.

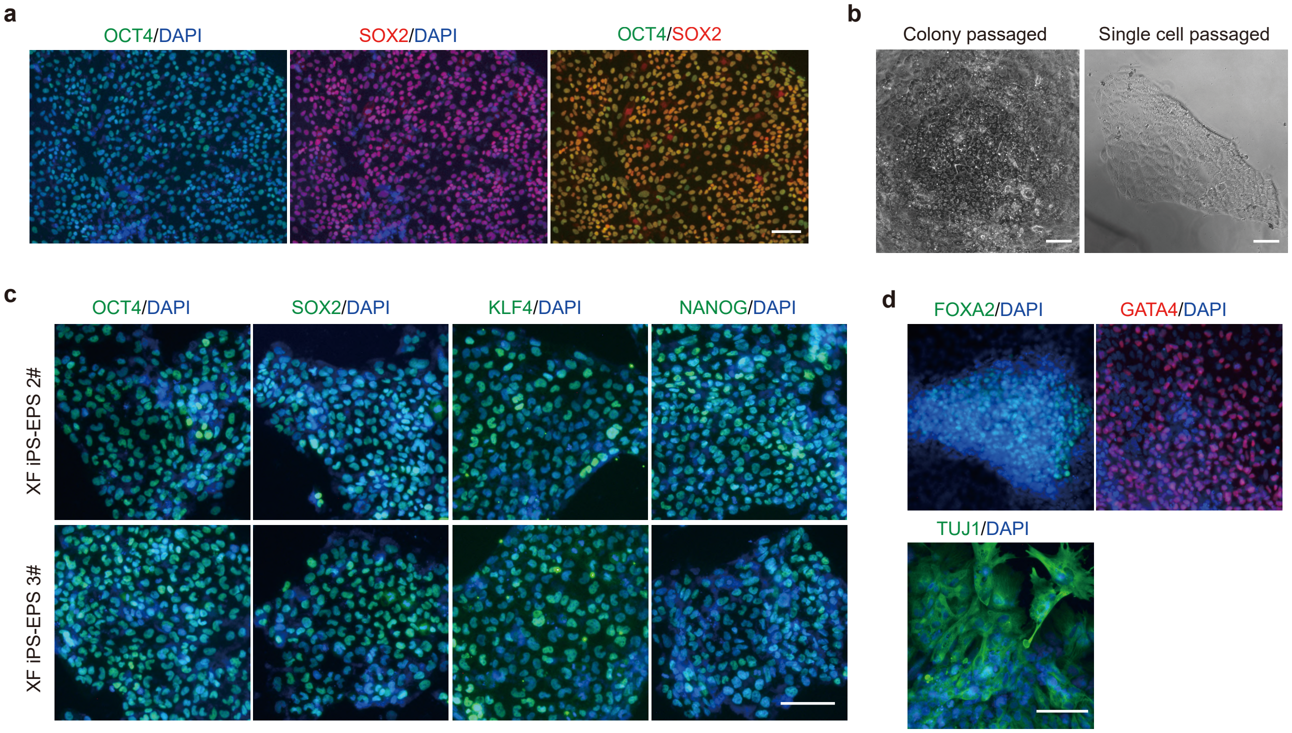

Supplementary Fig. 8. Generation of xeno-free human EPS cells from human fibroblasts by reprogramming.
a Representative immune-staining images showing OCT4 and SOX2 expression in picked hEPS-like colonies derived from human fibroblasts. Similar images were obtained in at least 3 independent experiments. **b** Representative images showing morphology of hEPS colonies derived from picked colonies and seeded single cells. Similar images were obtained in at least 3 independent experiments. **c** Representative immuno-staining images showing pluripotent marker gene expression in xeno-free hEPS cells derived from human fibroblasts. Similar images were obtained in at least 3 independent experiments. **d** Representative immune-staining images showing differentiated marker expression in xeno-free hEPS derivatives after EB differentiation. xeno-free hEPS cells are derived from human fibroblasts. Similar images were obtained in at least 3 independent experiments. Scale bars, 100 μm. Experiments in (a-d) were all independently repeated at least two times with similar results.

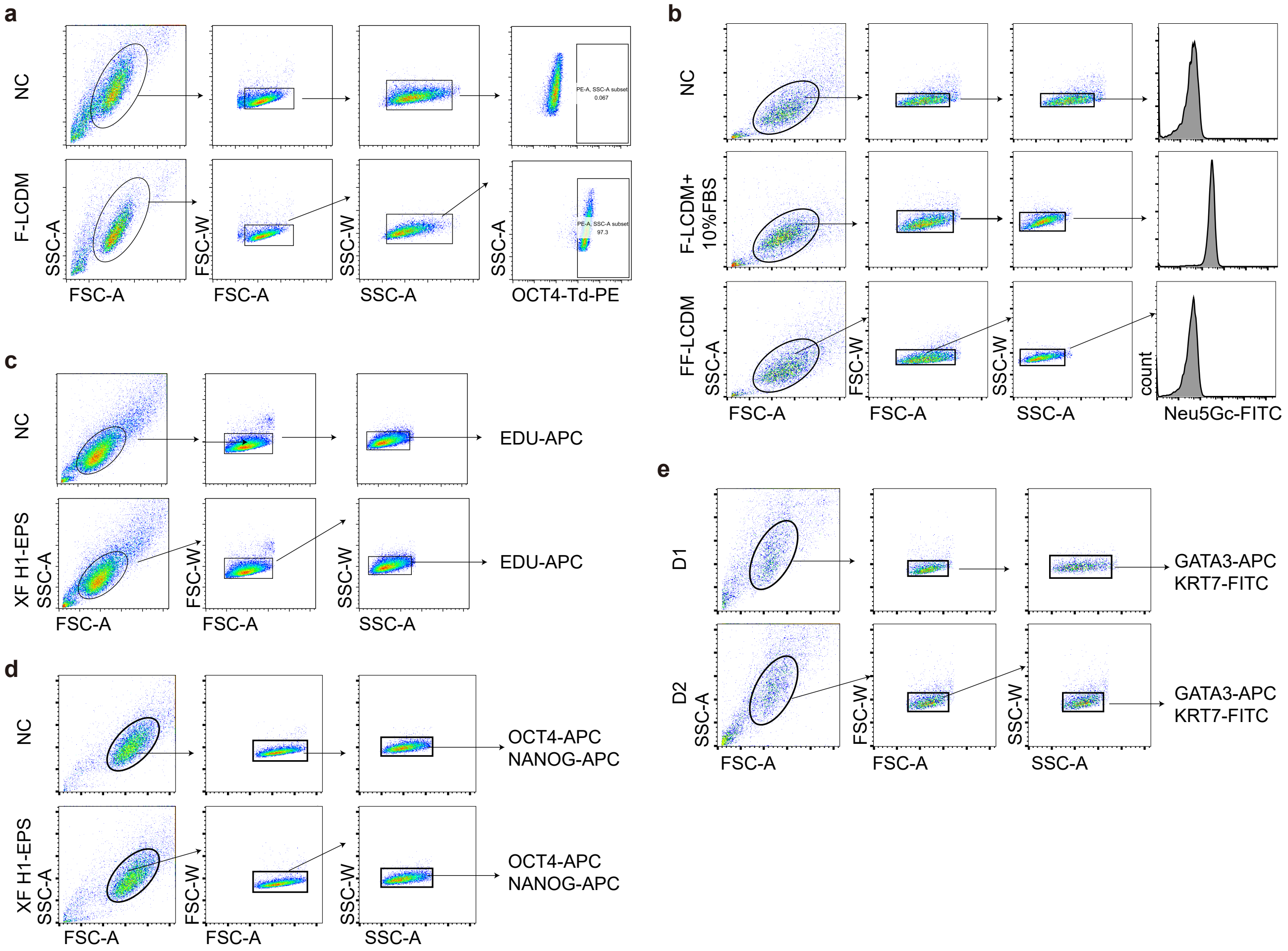

Supplementary Fig. 9. Gating strategies used for cell sorting.

a Representative images of gating strategy to sort OCT4+ cells from different culture conditions presented on Figure. 1a. **b** Representative images of gating strategy to sort Neu5Gc positive cells under different conditions presented on Supplementary Figure 2c. **c** Representative images of gating strategy to sort EDU+ cells from feeder cultured and xeno-free hEPS cells presented on Figure 2b and Supplementary Figure 3a. **d** Representative images of gating strategy to sort OCT4+ or NANOG+ cells from xeno-free hEPS cells presented on Figure 3a. **e** Representative images of gating strategy to sort KRT7+ and GATA3+ cells from xeno-free hEPS derivatives during trophoblast differentiation presented on Supplementary Figure 6d.

Supplementary References

1. Liu X*, et al.* Reprogramming roadmap reveals route to human induced trophoblast stem cells. *Nature* **586**, 101-107 (2020).

2. Petropoulos S*, et al.* Single-cell RNA-seq reveals lineage and X chromosome dynamics in human preimplantation embryos. *Cell* **165**, 1012-1026 (2016).

3. Xiang L*, et al.* A developmental landscape of 3D-cultured human pre-gastrulation embryos. *Nature* **577**, 537-542 (2020).

Supplementary Table 1: Media components of feeder free EPS medium

**Components**	**Concentration**	**Catalog Number**
DMEM/F12 (liquid)	2x	11330-032 (ThermoFisher Scientific)
Neurobasal (liquid)	2x	21103-049 (ThermoFisher Scientific)
Insulin	10 μg/ml	91077C (Sigma)
Apo-transferrin	5.5 μg/ml	T1147 (Sigma)
Sodium selenite	1 ng/ml	S5261 (Sigma)
Ethanolamine (liquid)	20000x	398136 (Sigma)
Catalase (liquid)	5000x	C3556 (Sigma)
L-Ascorbic acid 2-phosphate	100 μg/ml	A8960 (Sigma)
Activin A	5-40 ng/ml	HST-A-1000 (Stemimmune LLC)
Human LIF	10 ng/ml	300-05 (Peprotech)
CHIR99021	1 μM	S1263 (Selleck)
(S)-(+)-Dimethindene maleate	2 μM	1425 (Tocris)
Minocycline, Hydrochloride	2 μM	sc-203339 (Santa Curz)
Y-27632	5 μM	S1049 (Selleck)
**Optional components:**		
IWR-1-endo	0.5 μM	S7086 (Selleck)

Supplementary Table 2: Summary of extended pluripotent stem cells maintained in XF-LCDM

**Cell lines**	**Source**	**IF**	**EB**	**Teratoma**	**Karyotype**	**Long time culture**	**Chimera**
H1-EPS	H1	Yes	Yes	Yes	Yes	>70 passages	-
OT H1-EPS	H1	Yes	Yes	Yes	Yes	>70 passages	-
ES1-EPS	blastocyst	Yes	Yes	Yes	Yes	>50 passages	Yes
ES2-EPS	blastocyst	Yes	Yes	Yes	Yes	>50 passages	Yes
iPS-EPS	fibroblast	Yes	Yes	Yes	Yes	>50 passages	-
iPS-EPS 1#	fibroblast	Yes	Yes	Yes	Yes	>40 passages	Yes
iPS-EPS 2#	fibroblast	Yes	Yes	Yes	Yes	>30 passages	-
iPS-EPS 3#	fibroblast	Yes	Yes	Yes	Yes	>30 passages	-
iPS-EPS 4#	fibroblast	Yes	Yes	-	-	>30 passages	-
iPS-EPS 5#	fibroblast	Yes	Yes	Yes	Yes	>30 passages	-
iPS-EPS 6#	fibroblast	Yes	Yes	Yes	Yes	>30 passages	-
iPS-EPS 7#	fibroblast	Yes	Yes	Yes	Yes	>30 passages	-

Supplementary Table 3: Antibodies used for immunofluorescence and flow cytometry analysis

Antibody	IF Dilution	FACS Dilution	Catalog Number
Anti-OCT4	1:500	1:1000	611203 (BD)
Anti-OCT4	1:500		MA5-14845 (ThermoFisher Scientific)
Anti-OCT4	1:500		ab181557(Abcam)
Anti-NANOG	1:200		ab21624 (Abcam)
Anti-KLF4	1:200		ab75486 (Abcam)
Anti-SOX2	1:200		09-0024 (Stemgent)
Anti-rH2AX		1:1000	16-202A (Millipore)
Anti-GATA3	1:200	1:1000	ab199428 (Abcam)
Anti-KRT7	1:200	1:1000	M7018 (Dako)
Anti-HLA-G	1:200		ab7758 (Abcam)
Anti-hCG	1:500		ab9376 (Abcam)
Anti-FOXA2	1:200		ab108422 (Abcam)
Anti-SOX17	1:200		AF1924 (R&D)
Anti-GATA4	1:200		AF2606 (R&D)
Anti-GATA6	1:200		AF1700 (R&D)
Anti-TUJ1	1:500		ab107216 (Abcam)
Anti-NESTIN	1:200		MAB1259c (R&D)
Anti-CDX2	no dilution		Mu392a (Biogenex)
Anti-GFP	1:2000		ab13970 (Abcam)
Anti-CK18	1:200		ab668 (Abcam)
Anti-CK8	1:200		HPA049866 (Sigma)
Anti-Neu5Gc		1:1000	146901 (Biolegend)
Anti-NR2F2	1:100		ab211776 (Abcam)
Anti-XIST	1:50		SMF-2038-1 (Biosearch Technologies)

Supplementary Table 4: Primers used for mRNA and DNA expression detection

Gene		Primers
hGAPDH	F	TGACATCAAGAAGGTGGTGAAGCAGG
	R	GCGTCAAAGGTGGAGGAGTGGGT
hOCT4	F	GTGTTCAGCCAAAAGACCATCT
	R	GGCCTGCATGAGGGTTTCT
hNANOG	F	TTTGTGGGCCTGAAGAAAACT
	R	AGGGCTGTCCTGAATAAGCAG
hSOX2	F	GGGAAATGGGAGGGGTGCAAAAGAGG
	R	TTGCGTGAGTGTGGATGGGATTGGTG
hKRT7	F	AAGAACCAGCGTGCCAAGT
	R	TCCAGCTCCTCCTGCTTG
hHLA-G	F	CCACCACCCTGTCTTTGACTAT
	R	ACGTCCTGGGTCTGGTCCT
hCGB	F	GCTACTGCCCCACCATGACC
	R	ATGGACTCGAAGCGCACATC
hELF5	F	GACGCTGAAGAAAGCAAGGC
	R	CCCATTCCAGAATGCCACAG
hGATA3	F	TGCAGGAGCAGTATCATGAAGCCT
	R	GCATCAAACAACTGTGGCCAGTGA
hACTIN	F	GACAGCAGTCGGTTGGAGCG
	R	GGGACTTCCTGTAACAACGCATC
hCGβ	F	AGCACTTTGCTCGGGTCACGG
	R	TGGTCCAGCGCCAAGGGTGA
hTFAP2C	F	TGCACGATCAGACAGTCATTC
	R	GTAGAGCTGAGGAGCGACAATC
AAVS1-Clover CK1	F	CATCCTCCTTCCCCGTTGCCA
	R	CCGTGGGCTTGTACTCGGTCATC
AAVS1-Clover CK2	F	AACAAGCGCTCGACCATCACCTC
	R	AACCCCAGCCCACCCCAATC
Human-specific mitochondrial element	F	CGGGAGCTCTCCATGCATTT
	R	GACAGATACTGCGACATAGGGT
Human-mouse conserved mitochondrial element	F	GCTAAGACCCAAACTGGGATT
	R	GGTTTGCTGAAGATGGCGGTA
