# Supplementary material for: Chemically defined and xeno-free culture condition for human extended pluripotent stem cells
Source: Nat Commun. 2021 May 21;12:3017. doi: 10.1038/s41467-021-23320-8 (PMC8139978; doi:10.1038/s41467-021-23320-8)
Supplement: Supplementary file 5 — Description of Additional Supplementary Files [file 41467_2021_23320_MOESM5_ESM.pdf]

Title: Supplementary Data 1:
Description: Summary of whole genome sequencing EPS cell lines

Title: Supplementary Data 2:
Description: GO term analysis of ATAC-seq peaks in xeno-free and feeder-cultured hEPS cells

Title: Supplementary Data 3:
Description: Potential motifs for transcription factors in xeno-free and feeder cultured hEPS cells
